# Editorial: History of Chemoattractant Research

**DOI:** 10.3389/fimmu.2015.00548

**Published:** 2015-10-26

**Authors:** Bernhard Moser

**Affiliations:** ^1^Institute of Infection and Immunity, Cardiff University School of Medicine, College of Biomedical and Life Sciences, Cardiff University, Cardiff, UK

**Keywords:** history, chemokines, chemoattractants, migration, immunity

This Research Topic entitled “History of Chemoattractant Research” collects a series of personal stories by numerous experts in the field of chemoattractant research. The individual contributions portray some key discoveries that helped to transform cell migration research into a global playing field within immunology (and beyond). Early progress had a profound effect on both academia and industry. Today, numerous academic laboratories are fully engaged in compiling a detailed road map describing the highly complex network of immune and tissue cells that respond to chemoattractants. Industrial research, on the other hand, centers on drugs that interfere with immune cell traffic in inflammatory diseases and cancer.

By definition, chemoattractants include early (“classical”) chemoattractants of variable chemical composition and the large family of chemokines (chemotactic cytokines) that greatly outnumber the former compounds. As inferred from their name, all chemoattractants share the ability to induce cell migration (chemotaxis) via binding to a single class of G-protein-coupled receptors on target cells. Chemoattractant research was originally viewed as a specialty subject within cell biology. However, due to the increasing number of chemoattractants being discovered and their effect on every type of immune cells distributed throughout our body, it became quickly clear that chemoattractants constitute essential regulators of all aspects in immunity. Defects in the chemoattractant system are frequently associated with immunodeficiencies or autoimmunity/chronic diseases. We now know that the complexity of the chemokine and classical chemoattractant system perfectly mirrors the multitude of immune cells distinguished by lineage relationship, function, and tissue location. In fact, chemokine receptor profiling turned out to be highly useful for defining immune cell subsets as exemplified by the numerous T-helper subsets that we know today. Indeed, such work has led to a fundamental paradigm linking the functional specialization of distinct immune cells with their migratory behavior. No doubt, the principal and unifying function of chemoattractants is their ability to induce directional cell migration, involving processes as complex as immune cell transendothelial migration as well as chemokine gradient-controlled immune cell migration within tissues. In addition, some chemokines are able to costimulate T-cell differentiation, promote immune cell survival, or act as antimicrobial peptides in peripheral epithelial tissues. A few constitutive chemokines are essential for organ development during embryogenesis and some of these even control tumor cell relocation to secondary sites. Their importance is further emphasized by the realization that viruses have hijacked host genes encoding chemokines and their receptors in order to interfere with antiviral immunity or have evolved to use certain chemokine receptors as entry coreceptors.

The following series of “short stories” provide personal accounts on key discoveries. The individual molecular discoveries enabled numerous research laboratories worldwide to unravel their significance in steady-state or pathological immune processes. Although groundbreaking in their own right, it is worth emphasizing that rapid progress in chemoattractant research was only made possible by many other laboratories whose work attached “meaning” to these early findings. The authors of this miniseries are discussing their findings in the context of time, place, and subsequent progress enabled by their discoveries. It is hoped that a wide readership will find these accounts entertaining as well as educational although those who wish to gain a more detailed knowledge are referred to the many outstanding reviews on chemokines and other chemoattractants.

The field of chemokines really started in 1987 with the cloning of the human gene encoding CXCL8, which occurred in parallel in the laboratories of five independent international groups. Two stories, one by Marco Baggiolini (1) and the other by Teizo Yoshimura (2), summarize this groundbreaking discovery and give a vivid account about the friendly race that ensued from the realization that activated monocytes secreted neutrophil-specific chemoattractant activity to the molecular discovery of CXCL8. Unfortunately, and probably due to the enthusiasm shared by the research community at that time, it was decided to call CXCL8 an interleukin (IL-8), which turned out to be a misleading denomination. The three-dimensional structure of CXCL8 is a hallmark of all members of the chemokine superfamily and indicated that, in fact, chemokine-like proteins have been identified several years before CXCL8. These include IP10 (CXCL10) (3), LD78 (CCL3) (4), and TCA3 (5), the mouse ortholog of human I-309 (CCL1) (6). However, their chemoattractant activity remained obscure until well after the discovery of CXCL8. Also, platelet factor 4 (CXCL4) (7–9), the first peptide featuring a prototypical chemokine fold, was actually never shown to be a chemoattractant. The identification of CXCL8 immediately initiated a highly competitive search for its receptor(s) and the receptors for the well-described classical chemoattractant agonists, including the formylated bacterial peptide fMLP and the complement protein C5a. Norma and Craig Gerard summarize these early events from their own, personal perspective (10). By the early 1990s, the new field of chemokines took off in unprecedented speed, and it became quickly clear that the newly discovered chemokines not only targeted neutrophils but also monocytes and many other innate cells and even T and B cells. Tim Williams tells the exciting story about the discovery of eotaxin (CCL11) and its involvement in eosinophil recruitment during allergic diseases (11). Chemokines are implicated not only in infections and inflammatory diseases but also in homeostatic processes. The first such chemokine is SDF-1 (CXCL12), and Takashi Nagasawa tells his story about the importance of SDF-1 in embryogenesis, hematopoiesis, and even HIV infection (12). Unlike SDF-1, most homeostatic chemokines do not display a lethal phenotype in gene-deficient mice yet play an essential role in the traffic control of immune cells. Early work with orphan chemokine receptors in mice provided a first indication of the importance of chemokine receptors, notably BLR1 (CXCR5) (13) and BLR2 (CCR7) (14), in controlling cellular interactions within secondary lymphoid tissues (lymph nodes and spleen). I tell the story about how we at the Theodor-Kocher Institute and Martin Lipp’s group in Berlin identified CXCR5 as the specific marker for the novel T-helper cell subset termed follicular B helper T (T_FH_) cells (15). Not all chemokine receptors are capable of mediating cell migration responses, and these “non-signaling” receptors are now collectively called atypical chemokine receptors (ACKR). Three of these, ACKR1, ACKR2, and ACKR3, with unique functional features are subject of extensive investigations. ACKR3, previously known as RDC1 or CXCR7, binds CXCL12 with higher affinity than its primary receptor CXCR4 and, in addition, binds also CXCL11, one of the three CXCR3-specific chemokines (16, 17). ACKR3-deficient mice die *in utero* (18), suggesting a vital role in embryogenesis similar to what has been reported for CXCR4. Richard Horuk tells the story about ACKR1, also known as DARC or Duffy Antigen on red blood cells (19). It is an entry receptor for the malaria parasite *Plasmodium vivax* and, surprisingly, acts as a transcellular transporter of CXCL8 and many other chemokines in endothelial cells. The story about ACKR2, also known as D6, is told by Gerard Graham (20) and highlights yet another facet in chemokine research, namely the modulation of inflammatory milieus by ACKR2 via binding, uptake, and intracellular degradation of inflammatory chemokines. Chemokines do not act like normal cytokines do. In fact, immune cells expressing the corresponding chemokine receptors need to sense a chemokine gradient, and Amanda Proudfoot highlights the importance of glycosaminoglycans present on extracellular matrices in this process (21). The story by Paolo Lusso (22) describes how his groundbreaking discovery led to the immediate fusion of two seemingly unrelated fields of research, “chemokines” and “HIV infection,” fostering unprecedented collaborations between many international laboratories. His discovery of CCL3, CCL4, and CCL5 that acted as HIV-suppressor factors demonstrated that certain chemokines and possibly their receptors were involved in HIV infection. Almost simultaneously, fusin was reported to be the first HIV coreceptor, and this story is told by Edward Berger (23). Fusin turned out to be identical with the orphan chemokine receptor LESTR that we have published previously. In collaboration with Conrad Bleul, we then “deorphanized” LESTR by showing that this new chemokine receptor (CXCR4) is specific for CXCL12. Fernando Arenzana-Seisdedos summarizes these events and tells the story about CXCL12 and its HIV-suppressor activity (24). CXCR4 is not the only HIV coreceptor. Indeed, CCL3, CCL4, and CCL5, the HIV-suppressor factors, previously discovered by Paulo Lusso do not bind to CXCR4. The story by Marc Parmentier fills this gap and reveals that several groups worldwide, including his own, discovered CCR5 as the specific receptor for CCL3, CCL4, and CCL5 (25). CCR5 is the coreceptor primarily involved in person-to-person transmission of HIV, and individuals lacking CCR5 are largely protected against HIV. In clear contrast to HIV, many viruses carry genes that target the chemokine system, encoding either inhibitors that interfere with the function of chemokine receptors present on host immune cells or chemokine-neutralizing proteins with similarities to chemokine receptors, and Philip Murphy’s story touches on this important aspect of chemokine research (26). Chemokines and their receptors play a crucial role not only in viral diseases but also in all other inflammatory diseases as well as cancer. It is, therefore, obvious that chemokine receptors were selected as primary targets in translational research. Detailed structural data of chemokine receptors are of paramount importance for the design of small-molecular-weight inhibitors, and Tracy Handel’s story tells about the difficult journey she undertook to accomplish a high-resolution crystal structure of CXCR4 (27). Despite incredible investments by all major drug companies (as well as many small start-up businesses), the yield of approved chemokine receptor-specific drugs is still modest. In fact, the two success stories about FDA-approved compounds are not related to the treatment of inflammatory diseases. The first one by Elna van der Ryst tells the development of Maraviroc, a CCR5 antagonist used to treat HIV-infected individuals (28), and the second one by Erik de Clerk summarizes the discovery of the CXCR4-specific inhibitor AMD3100 and its use in hematopoietic stem cell mobilization (29).

Chemokine research goes on unabated although the race of molecular discoveries as highlighted here has well past its zenith. All major activities are now focused on understanding what all these original findings really mean. The field has progressed along so many different and seemingly unrelated routes that writing comprehensive reviews that cover all aspects of chemokine research has become a monumental task. One thing is certain, however, the last two decades have demonstrated once and for all that chemoattractant research can no longer be considered a subspecialty of cell biology.

## Conflict of Interest Statement

The author declares that the research was conducted in the absence of any commercial or financial relationships that could be construed as a potential conflict of interest.
